# Quantum inspired community detection for analysis of biodiversity change driven by land-use conversion and climate change

**DOI:** 10.1038/s41598-021-93122-x

**Published:** 2021-07-12

**Authors:** Sana Akbar, Sri Khetwat Saritha

**Affiliations:** Department of CSE, MANIT, Bhopal, India

**Keywords:** Climate sciences, Ecology, Engineering, Mathematics and computing, Physics

## Abstract

Community detection remains little explored in the analysis of biodiversity change. The challenges linked with global biodiversity change have also multiplied manifold in the past few decades. Moreover, most studies concerning biodiversity change lack the quantitative treatment central to species distribution modeling. Empirical analysis of species distribution and abundance is thus integral to the study of biodiversity loss and biodiversity alterations. Community detection is therefore expected to efficiently model the topological aspect of biodiversity change driven by land-use conversion and climate change; given that it has already proven superior for diverse problems in the domain of social network analysis and subgroup discovery in complex systems. Thus, quantum inspired community detection is proposed as a novel technique to predict biodiversity change considering tiger population in eighteen states of India; leading to benchmarking of two novel datasets. Elements of land-use conversion and climate change are explored to design these datasets viz.—Landscape based distribution and Number of tiger reserves based distribution respectively; for predicting regions expected to maximize Tiger population growth. Furthermore, validation of the proposed framework on the said datasets is performed using standard community detection metrics like—Modularity, Normalized Mutual Information (NMI), Adjusted Rand Index (ARI), Degree distribution, Degree centrality and Edge-betweenness centrality. Quantum inspired community detection has also been successful in demonstrating an association between biodiversity change, land-use conversion and climate change; validated statistically by Pearson’s correlation coefficient and *p* value test. Finally, modularity distribution based on parameter tuning establishes the superiority of the second dataset based on the number of Tiger reserves—in predicting regions maximizing Tiger population growth fostering species distribution and abundance; apart from scripting a stronger correlation of biodiversity change with land-use conversion.

## Introduction

Over the last few years, topological analysis of social networks has garnered much attention in diverse applications. Most of these studies define social networks to represent a skeletal interpretation of social interactions governed by the study of concurrently occurring vertices and edges^1^. SNA or social network analysis; thus allows us to extract ideas, typical patterns, or archetypes that are of specific use to the concerned group or organization. In this regard, community detection plays a crucial role in unearthing common features within any social network; enabling its clear division and sharp visualization^2^. Moreover, identification of distinct aspects of a community structure through their membership criterion, inter and intra community roles, weighted or unweighted links, the extent of overlap, presence or absence of hierarchy^3,4^, etc.; ensures enhanced visibility and localization of underlying cohesive subgroups^2^.

Consequently, community detection has emerged as a promising technique for the analysis of information flow using computational frameworks in the study of complex networks^5^. Ecological network analysis has been applied successfully in solving numerous problems in studying biodiversity change^6–8^. This has directed the focus of the scientific community towards the applicability of community detection techniques in the study of biodiversity change. As we know, biodiversity change has been the area of interest for researchers and environmentalists for quite a while now^9,10^. The study of biodiversity change using species distribution modeling has gathered much attention recently due to the magnitude of its implications observed in the current times^11^. Thus, biodiversity change is broadly categorized into—biodiversity loss and biodiversity alterations; spanning over four dimensions of biodiversity viz.—species extinctions, species abundances, species distributions, and genetic diversity^12^.

However, most treatments on biodiversity change so far have been based on subjective, descriptive and qualitative analysis^6–12^. There are too many intangible considerations and too many pitfalls in the conventional wisdom of economics. Therefore even after years since biodiversity change became a serious concern impeding the realization of sustainable development, the attained progress has not been enough to curtail its imminent risk^13,14^. Firstly, the taxonomic coverage related to biodiversity change is limited due to little knowledge about the vast majority of biodiversity facing extinction risk^12^. This reinstates the need for employing dynamic and network agnostic methods for predicting biodiversity change. Secondly, the format and storage of data related to biodiversity change is not consistent across different architectures^6^. This calls for architecture-independent and portable systems that could leverage the vast incoherent data disintegrated across incompatible platforms. Thirdly, the pressing demand for a context-based and ontologically richer representation of species abundance and distribution^6^ requires capturing network interactions in addition to the spatial or temporal information of the underlying network. Fourthly, a more robust framework is essential to predict from sample data in both weighted and unweighted networks; for better comprehension of species-environment interactions^15^.

In this regard, quantum inspired community detection has emerged as a viable option to limit the uncertainties in designing species distribution models (SDMs) by ensuring—quantum parallelism, exponential speedup and portability, dynamic allocation of cluster size and architecture, efficient modeling of metadata and data-level interactions, reduced parameter dependency, etc. (Table 1). The paper thus aims to facilitate improved network analysis by integrating most recent technologies like Quantum inspired machine learning (QIML) based community detection—as a novel tool manoeuvring biodiversity change. QC based algorithms have already been found relevant in—“handling NP hard problems employing community detection due to their intrinsic ability of evolving dynamically with qubits and easy translation whilst being implemented on quantum devices”^16–19^. Moreover, the transcendence of QML (sprouting from the merger of QC with ML) based community detection “is established from its capacity of building a new class of quantum easy problems (BQP) falling under NP class for classical algorithms”^20^. Therefore, most work in the field of quantum inspired CD has been done by applying QML based algorithms to complex systems; registering better performance than standard classical CD methods.

**Table 1:** Summary of quantum inspired community detection methods.

Methodology	Principle idea	Advantages	Limitations	References
Quantum inspired evolutionary algorithm (BinQIEA)	It is based on a local-search model with modularity maximization aimed community detection	It sorts subgroups in LFR and real world networks not requiring knowledge of cluster size and architecture	Its applicability in identifying communities in overlapping structures is still unverified-Computational time can be improved	2014^62^
NumQIEA	It employs parameter tuning in swarm optimization based clustering	Shows better results than other variants of QIEA	Its computational time could be improved further in solving varied optimization problems	2014^63^
IMOQPSO	IMOQPSO could be understood as an enhanced form of multi-objective QPSO utilizing fine tuning for further refinement of the outcomes	line graph modeling is used for detection of overlapping communities in real world and synthetic networks	It extracts spectral density for detecting communities, thus being computationally intensive	2015^64^
Parallel version of Quantum inspired evolutionary algorithm	It is a HPC based implementation of QIEA enabling parallel processing through NVIDIA graphic card	It enables modest speedup due to incorporation of quantum parallelism in each chromosome	Its applicability in contributing to biological and overlapping networks is still anomalous	2017^65^
QDMPSO	It employs a discrete version of swarm optimization based clustering using uncertainty principle	It ensures reduced parameter dependency by MOP formulation and non-dominant sorting based modularity resolution	Scalability is still an obstacle in larger networks-The complexity ranges in the order of O ${(\mathrm{N}(\mathrm{m}+\mathrm{n}+\mathrm{N}}^{2}$)), which could be further improved	2017^66^
QIEA-net and iQIEA-net	It uses different probability & no. of guiding for QIEA to show how a single guiding quantum individual accelerates optimal convergence	It doesn’t require fine tuning of parameters and allows dynamic cluster allocation	The accuracy of detected communities could be improved Computational time needs to be improved	2018^67^
CD with diverse Quantum Architectures	A hybrid quantum classical framework is put forth to ensure architecture independent and portable system	Advocates NISQ hardware with minimum qubits for catering to large scale problems Ensures an extensible system by implementing a given CD algorithm with diverse quantum computing paradigms	Scalability of existing algorithms is still a distant dream-Access to a (UQC) or universal quantum computer is limited to a handful of scientists	2018^68^
Quantum Annealer	Designed and executed a CD problem on D-wave 2X quantum annealer recording significant improvement in computational time	Utilizes qbsolv software as a means to encode a QUBO problem, to minimize the problem globally making multiple calls to D-wave It inhibits the need to execute recursive processes; unlike its classical counterparts	Problem reformulation is needed for identifying three or more communities Current quantum systems have sparse connectivity, narrow precision, and less number of available quantum bits	2020^69^

Existing studies suggest how species response to various characteristics of land-use conversion and climate change vary considerably based on a number of factors^9–11,21^. Habitat change and climate change have been found to be two of the key drivers of biodiversity change^6^. Study and validation of correlation between biodiversity change and land-use conversion and climate change has emerged as a key objective governing biodiversity change^6,9,21^. The motivation of this paper is to provide the quantitative treatment central to species distribution modeling by—empirical analysis of species distribution and abundance using quantum inspired community detection. Community detection is therefore explored to efficiently model the topological aspect of biodiversity change driven by land-use conversion and climate change. Although, there are few studies that suggest the application of CD approaches for the analysis of networks related to climate change^22,23^ and meteorological data^24,25^. Yet to our knowledge, no work has been done so far that studies the applicability of community detection for biodiversity change.

As elaborated in^6^, the authors stressed the need for next-generation studies to comprehend the properties of network structure by employing graph theory based methods like—centrality, modularity, connectance, intervality, etc. for species interaction networks. Context-based analysis of network interactions built around amalgamation of empirical analysis with statistical proof is thus found to be better equipped at predicting underlying network structure and vice versa. Another study^26^ discusses the efficacy of using degree distribution based analysis of ecological networks. Accordingly, this novel study incorporating CD approaches for analysis of biodiversity change provides empirical validation based on standard CD metrics like-Modularity^27^, Normalized Mutual Information (NMI)^28^, Adjusted Rand Index (ARI)^29^, Degree distribution^30,31^, Degree-centrality^32^ and Edge-betweenness centrality^33^ on two novel datasets. There are several studies like^34,35^ that recommend the use of above mentioned metrics for performance analysis of community detection methods.

As already stated, land-use conversion has been found to be strongly correlated with biodiversity change^6,9,21^. Recent studies^36^ have also highlighted the correlation between land surface temperature (LST) and type of landscape; linking LST with land-use, land-cover change wrt effect of change in elevation. Also, landscape based classification has been effective in capturing heterogeneity and context in examining the response of surface temperature^37^. Similarly, another study^38^ links and evaluates the impact of landscape patterns on regional precipitation as a means to regulate climate forcings in biodiversity change. There have been few studies in the past that have focused on—studying climate change trends in India^39,40^ or accelerating Tiger conservation through Landscape genetics and Habitat linkages^41^; serving as a template for designing a framework intended for the study of biodiversity change. This forms the motivation for the design of datasets analyzing Tiger population in India viz.—Landscape based dataset and Number of tiger reserves based dataset; for predicting regions expected to maximize Tiger population growth. Anomalies in temperature and precipitation are mapped with detected communities as a means to correlate Landscape based distribution with biodiversity change; while the average percentage increase in the area of Tiger reserves and the increase in Tiger reserves are mapped with detected communities as a means to correlate the number of tiger reserves based distribution with biodiversity change—both characterized by a change in species abundance and distribution.

Quantum inspired community detection has thus been successful in demonstrating an association between biodiversity change, land-use conversion and climate change validated statistically by Pearson’s correlation coefficient and *p* value test. Finally, modularity distribution based on parameter tuning establishes the superiority of the second dataset based on the number of Tiger reserves—in predicting regions maximizing Tiger population growth fostering species distribution and abundance; apart from scripting a stronger correlation of biodiversity change with land-use conversion.

As a future initiative, other performance measures could be used to further validate the accuracy of detected communities like—(eigenvector centrality and closeness centrality^35^), purity^42^, fuzzy rand index^43^, etc. for overlapping as well as disjoint communities. Moreover, community detection approaches could be extended for analysis with other drivers and their interactions; ameliorating or exacerbating biodiversity change. Metrics like Community Temperature Index^44^ and Living Planet Index^45^ could also be explored to study species abundance change in the future.

## Materials and methods

As discussed in^46^, the design of rough planetary boundaries presents a holistic approach to global sustainability by defining safe operation limits for each quantified sustainability indicator. Transgressing over this limit may start a sudden disruptive change that might be difficult to undo. For instance, as per the analysis carried out in^46^, the permissible limit for—climate change, rate of biodiversity loss, and changes to the global nitrogen cycle has already been surpassed; thus causing an irreversible loss.

Thus, dedicated efforts are required to tackle the uncertainties associated with the study and modeling of biodiversity change, climate change, land-use alteration, etc. As discussed earlier, biodiversity change is broadly categorized into—biodiversity loss and biodiversity alterations^12^; spanning over four dimensions of biodiversity viz.—species extinctions, species abundances, species distributions, and genetic diversity (Supplementary Fig. 1). Similarly, climate change could be defined as—“A statistically significant trend of climate state on longer timescales (decades or more)^47^.” It could be classified primarily into—natural and anthropogenic (Supplementary Fig. 2). With the advent of climate modeling, it has now become possible to interpret physical aspects of climate system viz.—land, ocean and atmosphere; in the form of equation sets for energy, momentum and mass conservation. Additionally, climate models could be classified either into—coupled general circulation models (GCMs) that employ spatial discretization & are extremely computer intensive or simple climate models employing coarse spatial resolution that cater to a limited subset of physical processes. A new class of models with intermediate complexity has emerged recently to optimize the degree of complexity with computational cost. These intermediate models are found most appropriate in studying past climate changes for long-term prediction of future climate change. Furthermore, modeling land-cover change is expected to tackle varying spatial and temporal scales^48^; furthering the focus on local models to minimize the trade-off between different socio-economic factors^49^. These bottlenecks in the modeling and analysis of different drivers of biodiversity change; push the need to incorporate machine learning based techniques like quantum inspired community detection for empirical analysis of biodiversity change.

Thus, climate change driven landscape based dataset and land-use conversion driven Number of Tiger reserves based dataset are designed to predict biodiversity change across eighteen states harboring Tiger Reserves in India. The two novel datasets classify the 18 states into four sub-groups; taking the percentage increase in Tiger population recorded between 2010 and 2014 as the criteria for establishing intra-links (Supplementary Table 1 and Supplementary Table 2). Inter links are formulated for 18 states (excluding Goa and Nagaland for incomplete data) in two ways:

1. **Landscape based dataset:** Grouping the 18 states surveyed into 4 subgroups based on their geographical landscape. (Supplementary Table 1).
2. **Number of Tiger reserves based dataset:** Bifurcating the 18 states into 2 subgroups based on the no. of Tiger reserves present in each state. (Supplementary Table 3 and Supplementary Table 4).

This leads us to the benchmarking of two novel datasets in the form of Landscape based dataset and number of Tiger reserves based dataset; both drawn after establishing percentage increase based intra-links. The criteria for the identification of distinct subgroups thus form the basis of community detection in the analysis of complex networks. Accordingly, the percentage increase wise division of states with Tiger population for the year 2014 helps predict future trends; validated by the trend recorded for the year 2018 (Supplementary Table 1 and Supplementary Table 2).

As evident from Supplementary Table 2, This splitting of states considering percentage increase in Tiger population as the chosen parameter of interest for Landscape and Number of Tiger reserves based distribution; divulges hidden patterns associated with the given ecosystem that helps in prioritizing the sub-communities (states) showing better results (see “Results and discussion” section). It also goes a long way in defining the future trends as evident from the similar trend recorded for the year 2018 as shown in Supplementary Table 1.

As stated already, Landscape based distribution is used to classify inter-links based on the landscape a given state falls into; as shown in Supplementary Table 1. For the second dataset, we consider the number of Tiger reserves found in each state [https://bigcatsindia.com/tiger-census-2018/.] (Supplementary Table 3) and group them into 2 subgroups with the states with 3 or more reserves forming one class and the rest forming the second (Supplementary Table 4).

Before going for the empirical evaluation on two novel datasets, we briefly discuss the literature associated with community detection for the study of interactions governing biodiversity change^22–25^ and how the existing CD approaches could be applied for analysis of biodiversity change^50,53–57^.

As discussed in^22^, a method is proposed that enables community detection by incorporating details of the response variable leading to a graphical illustration of spatiotemporal data combined from different variables. It is found to perform better than existing methods used for climate index discovery linked with seasonal rainfall variability based on network analysis as well as statistical validation. Similarly, a community detection method^23^ is used to identify multivariate clusters based on cross-correlation assisted network weight assignment. It also offers a climatological interpretation of climate anomalies; devising a way to detect the disturbances efficiently. Additionally, a geographical location embedded community detection method^24^ is put forth to analyze climate data in meteorological networks. It further reveals the link of community structure with topographical and land-use related data and how climate change and land-use related data could be modeled topologically to decipher their underlying structure.

Lastly, a recent study^25^ has demonstrated how CD techniques could be altered to make them geographically sensitive by adding spatial weighting to the input flow network. It may also be used to study how the communities change over time (days, weeks, day, night, etc.) and by incorporating contextual information (temp., pollution, weather, etc.) to it. Adding geographical weighting to LOUVIAN algorithm^50^ boosts the number of detected communities from 3 to 14 while raising this number from 1(i.e. adding no new information about passenger commute) to 99 for hierarchical link clustering algorithm^51^ for a given sample graph^25^. This spatial classification works well for overlapping communities as well by allowing movement-based classification in addition to location-based classification.

Thus, integration of CD techniques with geographical weighting is expected to serve as an effective tool in designing Wildlife Corridors, to reunite misplaced animals with their flock; by analyzing the movement patterns of animals in locating high breeding areas and isolating the links acting as bridges across varied ecosystems, thereby promoting Habitat Preservation^25,52^. It not only safeguards endangered species but also offers a symbiotic solution for human coexistence with nature. Thus, geographical weighting based CD techniques that analyze movement patterns to assist habitat preservation would also check anthropogenic climate forcings.

Over the years, there are a number of standard CD algorithms developed like—LPROP^53^, LOUVIAN^50^, CNM^54^, N-eigen^55^, Walktrap^56^, GN fast^57^, etc. that have been evaluated on various parameters including their execution time, the effect of average network degree and the mixing parameters, memory requirement, scalability, etc. Although, most of these algorithms register good modularity or NMI values; yet they suffer from memory constraints imposed by the absence of quantum parallelism, inability to balance between local and global exploration and needing prior information about cluster size and architecture^4,27,52–57^. Also, modularity based comparative analysis of the proposed novel implementation of quantum inspired community detection algorithms against the std. CD approaches^50,53–57^ is illustrated in (Fig. 5 and Supplementary Fig. 3); where quantum inspired CD algorithms are found to outperform the std. CD algorithms.

This leads to the emergence of quantum inspired machine learning (QIML) based community detection to remove most of the bottlenecks associated with classical CD approaches. Existing studies demonstrate how quantum approaches like quantum walks and quantum transport clustering have been employed to locate marked nodes in a network demonstrating a complexity of O(√n) which is not achievable by classical algorithms^58,59^. A quantum approach in community detection could range from—detecting communities in quantum complex networks^60^, implementing CD techniques in small quantum computers^61^ to formulating quantum inspired machine learning techniques for community detection (Table 1).

As evident from Table 1, quantum inspired machine learning based CD algorithms like—(parallel and serial versions of binQIEA^62,65^, numQIEA^63,65^), IMOQPSO^64^, QDMPSO^66^, QIEA-net^67^, iQIEA-net^67^ and QA^69^ have evolved by the amalgamation of QC with ML techniques—giving birth to QML. QIML forms a class of QML that relies on quantum characteristics but is realized on classical machines, displaying the capacity for easy translation on a quantum machine. With an exception of QA^69^ and hybrid quantum classical framework^68^ that work on a quantum machine; all the above mentioned QML algorithms fall in the class of QIML algorithms realizable on classical machines.

Moreover, as discussed in^70,71^, quantum supremacy in the domain of community detection is established and validated by the modularity based comparative analysis for real world datasets; where quantum machine learning (QML) based CD algorithms (forming a superset for QIML) outperform the standard state of the art as well as classical ML based CD algorithms. Also, criteria for the selection of a suitable CD algorithm based on optimized memory usage, computational complexity, scalability, dynamic nature, etc. is a driving force for efficient network analysis as elaborated in^4,70^. All these factors play a crucial role in selecting CD methods for the analysis of any complex network including the networks governing biodiversity change. Accordingly, quantum inspired CD algorithms have been found to outperform most std. CD algorithms due to—quantum parallelism, dynamic allocation of cluster size and architecture, reduced parameter dependency, etc.

As already known, QML serves to supersede a set of NP hard problems by remodeling them into a class of quantum easy problems^20^. Quantum inspired machine learning (Supplementary Fig. 4) is thus, fated to prove advantageous for learning problems and in reducing the computational time of ML algorithms.

Additionally, the leap from classical to quantum is characterized by the translation from a bit to qubit. “A physical realization of a qubit makes use of both energy states of an atom: an excited level representing |1>, a ground level representing |0> and a superposition of both the states by being in the ground and excited state simultaneously. A single qubit could be constrained into a superposition of two states expressed by adding the state vectors mathematically:

1 $$\left| \Psi \right\rangle = \upalpha _{{\mathbf{1}}} \left| {\mathbf{0}} \right\rangle + \upalpha _{{\mathbf{2}}} \left| {\mathbf{1}} \right\rangle$$

where $\upalpha _{{\mathbf{1}}}$ and $\upalpha _{{\mathbf{2}}}$ are complex numbers satisfying the condition as given:

2 $$\left| {\upalpha _{{\mathbf{1}}} } \right|^{2} + \left| {\upalpha _{{\mathbf{2}}} } \right|^{2} = {\mathbf{1}}$$

In the previous Eq. (2), |$\left| {\upalpha _{1} } \right|^{2}$ denotes the possibility of the superposition collapsing to |0>.”

Let us consider the example of our two novel datasets to understand how quantum inspired algorithms operate on quantum characteristics to optimize modularity in the community detection process. A sequence forming initial network skeleton for two datasets comprising of 18 nodes, along with its transformation obtained by hierarchical bi-partitioning applied at each level is shown in Supplementary Fig. 5.

Furthermore, as the system evolves, measure operation causes each qubit to converge to either 1 or 0 according to the wave function collapse. If the mutated gene in P(t) boosts the network modularity, then the mutation is accepted. Similarly, quantum versions pertaining to diverse problems could be defined for all other classes of quantum inspired machine learning (QIML) based community detection algorithms.

Thus, we observe that the formulation of two novel datasets is followed by their empirical validation based on standard CD metrics like—degree centrality, edge-betweenness centrality and degree distribution. Accordingly, the novel benchmarked datasets are further evaluated based on the most commonly used performance measures like—modularity, NMI and ARI using Girvan Newman algorithm as a standard CD approach. Lastly, QIML based CD algorithms are implemented—to obtain modularity based distribution to compare the two datasets and for modularity based comparative analysis with Std. CD methods to cement the superiority of the proposed QICD approach for analysis of biodiversity change.

Lastly, Pearson’s correlation coefficient and *p* value test are introduced for statistical validation of correlations established between biodiversity change and land-use conversion or climate change. The framework used for the design and implementation of the proposed approach in the analysis of biodiversity change is explained in (Supplementary Fig. 6).

## Results and discussion

The network diagrams obtained for the two datasets viz.—Landscape based dataset and Number of Tiger reserves based dataset are plotted as shown in [Supplementary Fig. 7(a) and Fig. 7(b)] respectively.

**Figure 1:**
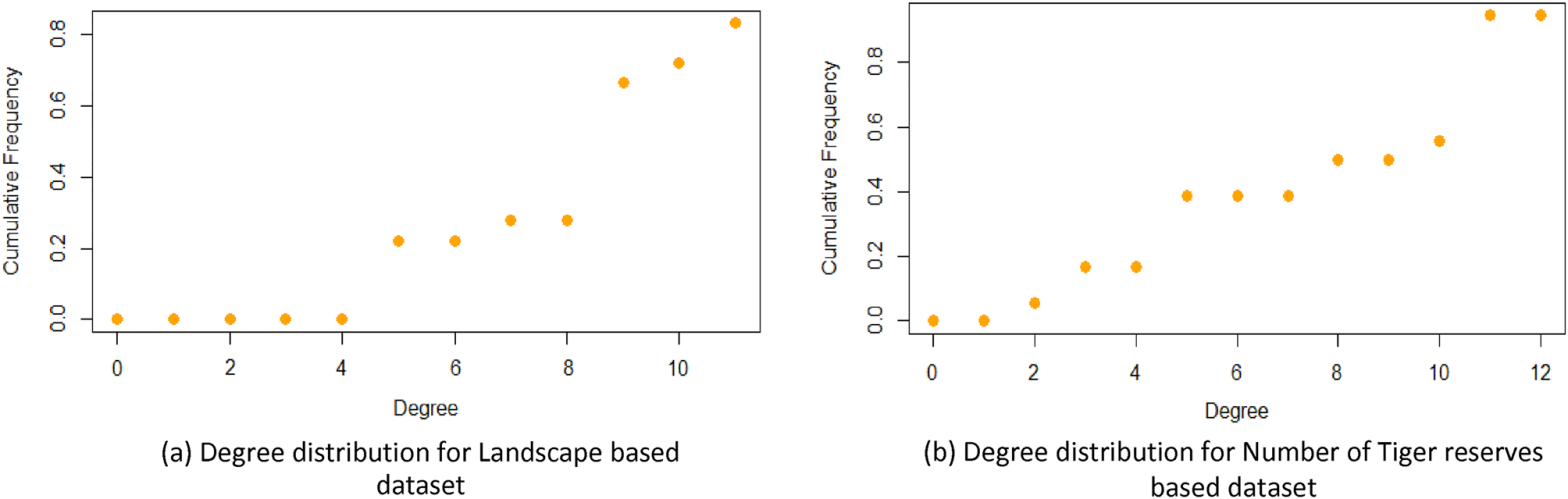
Degree distribution for Landscape based dataset and Number of Tiger reserves based dataset.

As evident from degree distributions^30,31^ of the two novel datasets as shown in Fig. 1a,b; the second dataset based on the number of Tiger reserves displays higher centralization of nodes (also observed in the network diagrams) thereby illustrating a more defined community structure.

**Figure 2:**
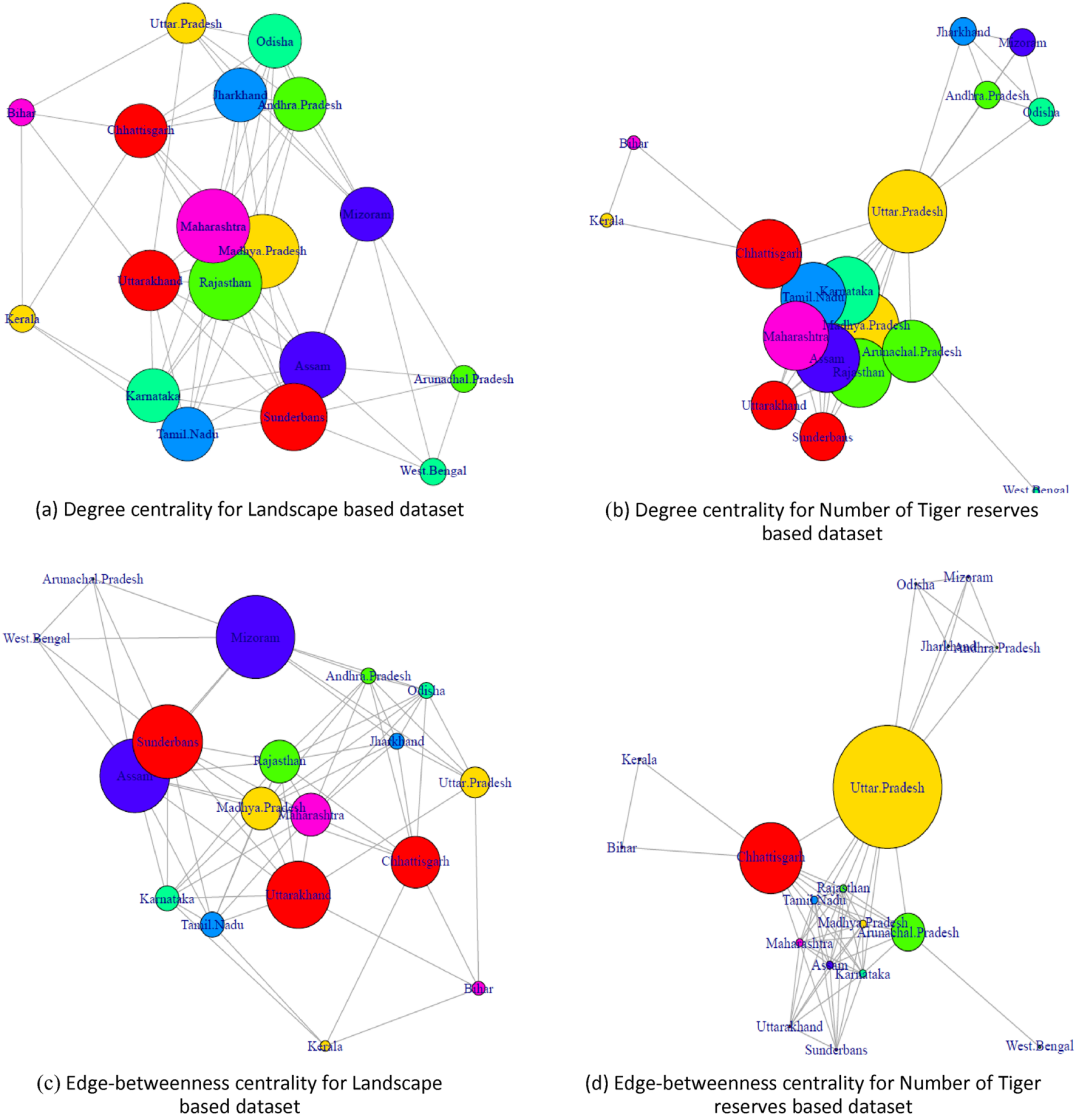
Degree and edge-betweenness centrality for Landscape based dataset and Number of Tiger reserves based dataset.

A comparative analysis based on centrality measures on the given datasets further proves the superiority of the second dataset based on the number of Tiger reserves in subgroup discovery for predicting more important nodes. Centrality based comparative analysis on given datasets is shown in Fig. 2.

Figure 2a,b show degree centrality based representation of individual nodes, while Fig. 2c,d show edge-betweenness based centrality based representation of individual nodes for Landscape based dataset and Number of Tiger reserves based dataset, respectively. The vertex size of each node is plotted as a function of its degree or edge-betweenness, thus highlighting the most important vertices based on degree centrality^32^ and betweenness centrality^33^.

Degree distribution^31^ for each node is shown for both datasets in Fig. 1, recording higher centralization of nodes for Number of Tiger reserves based dataset; advocating its supremacy in prioritization of states for Tiger conservation activities. Additionally, the importance of a node is ascertained by its degree centrality^32^ given by the number of nodes adjacent to it (the greater the number, the more crucial the node). As evident from Fig. 2a,b, the nodes with the highest degree centrality are determined more accurately by the Number of Tiger reserves based dataset. The second dataset assigns the highest degrees to five states that record maximum increase in Tiger population in 2018 with an addition of Uttar Pradesh (12) and Karnataka (11); including the only three states [Madhya Pradesh(11), Maharashtra(11) and Rajasthan(11)] determined by the first dataset. Also, it might be noted that Tamil Nadu, Assam and Uttarakhand fall in the next most important node category recording nominal growth for both datasets [node degree ranging between (7–10)]; while Chhattisgarh (10) and Arunachal Pradesh (9) mark the states of least priority for Tiger population growth related activities; recognized as crucial nodes (with higher DC) only by the second dataset. Thus, degree centrality based analysis of given datasets helped in identifying states likely to show a similar trend in the future and validated by the actual trend observed in 2018.

Similarly, edge betweenness is also calculated using betweenness centrality^33^; which counts the number of shortest paths that pass one node. As shown in Fig. 2c,d, edge betweenness is calculated using igraph package for—Landscape based dataset and Number of Tiger reserves based dataset, respectively. Nodes with high betweenness are important in communication and information diffusion. Landscape based dataset marks Mizoram (11.83), Sunderbans (10.52) and Assam (10.52) as the most crucial nodes based on edge-betweenness making them most suitable for edge-removal; enabling separation into subgroups. The number of Tiger reserves based dataset, on the other hand; assigns the highest edge-betweenness to Uttar Pradesh (52) followed by Chhattisgarh (30); leading to improved community division in line with degree centrality based analysis.

Additionally, subgroup discovery using Girvan Newman^72^ based community detection algorithm divides the two datasets viz.—Landscape based dataset and Number of Tiger reserves based dataset into four communities each; as shown in (Supplementary Fig. 8(a) and Fig. 8(b)] respectively.

**Figure 3:**
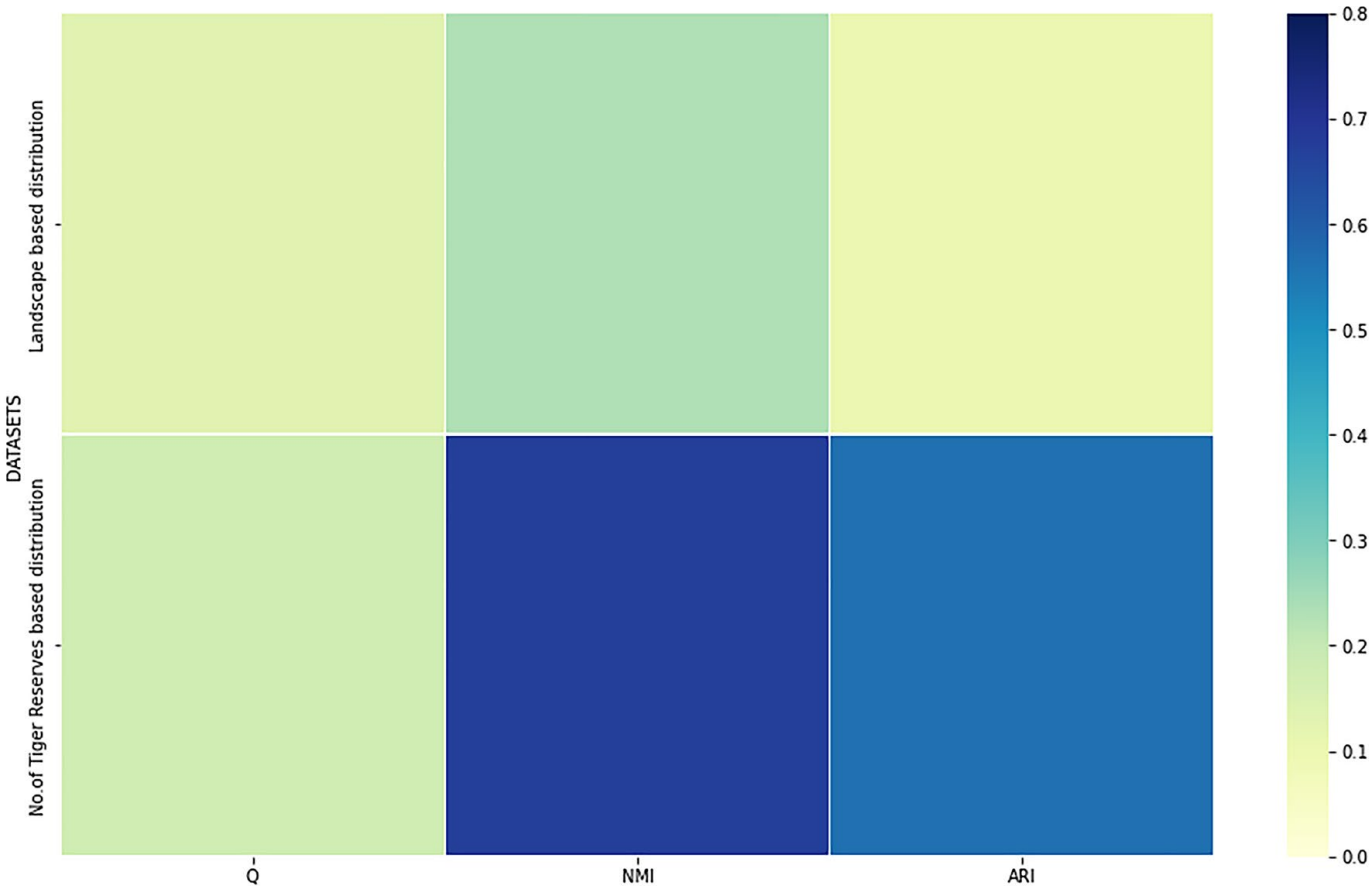
Modularity (Q), NMI and ARI based numerical analysis of Landscape based dataset and Number of Tiger reserves based dataset.

A heatmap based illustration of the numeric analysis is drawn using python 3.8 for performance measures like—modularity (Q), NMI and ARI as given in Fig. 3. This further cements the effectiveness of the partitioning undertaken in the form of four communities for each dataset.

As evident from the heatmap, the second dataset based on the number of Tiger reserves records better performance with modularity (Q) [0.177], NMI [0.6749] and ARI [0.56459] values obtained as compared to modularity (Q) [0.133], NMI [0.2327] and ARI [0.1058] values recorded for Landscape based dataset. Consequently, not only have we benchmarked two novel datasets for the application of community detection algorithms in the analysis of growth trends in the Tiger population; but have also been able to assess their performance by centrality measures and empirical analysis. This goes a long way in the identification and selection of requisite parameters of interest for the design of complex networks, including species distribution models.

**Figure 4:**
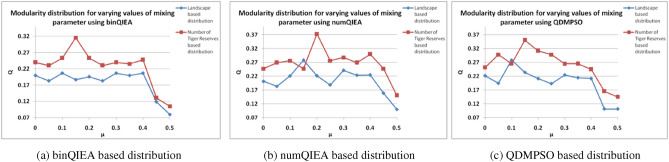
Comparative modularity (Q) based distribution of QIML approaches for varying values of mixing parameter (µ) for two novel datasets.

Lastly, QIML based CD is explored for further validation of the efficacy of the two novel datasets in capturing the structure of the network analyzing biodiversity change. It has also been observed that parameter tuning plays a prominent role in determining the efficiency of any evolutionary algorithm as shown in Table 2. The best values obtained corresponding to each dataset for the three QIML based CD algorithms have been highlighted in bold as shown in Table 2 while the best values obtained corresponding to each dataset using Pearson's r-value test for statistical analysis have been highlighted in bold as shown in Table 3. Parameters are tuned as per NSGA-II^73^ with standard values of ${{\uptheta }}_{1}$, ${{\uptheta }}_{2}$, ${\upalpha }$ and ${\upbeta }$; with varying values of mixing parameter (µ) leading to comparative modularity distribution for the two datasets as shown in Fig. 4.

**Table 2:** Modularity based analysis of QIML based CD algorithms for varying values of mixing parameter—for Landscape based and Number of Tiger reserves based datasets.

Datasets	Nodes	Edges	NC	µ (Mixing parameter)	Modularity (Q)		
					binQIEA	numQIEA	QDMPSO
Landscape based distribution	18	70	4	0.0	0.1996	0.2017	0.2218
			4	0.05	0.1832	0.1834	0.1946
			4	0.1	0.**2068**	0.2215	**0.2787**
			4	0.15	0.1865	0.**2784**	0.2337
			4	0.2	0.1957	0.2219	0.2114
			4	0.25	0.1832	0.1887	0.1932
			4	0.3	0.2066	0.2411	0.2244
			4	0.35	0.1995	0.2237	0.2145
			4	0.4	0.2065	0.2248	0.2119
			4	0.45	0.1188	0.1587	0.1014
			4	0.5	0.0799	0.0999	0.1012
Number of Tiger Reserves based distribution	18	63	4	0.0	0.2397	0.2474	0.2513
			4	0.05	0.2304	0.2689	0.2984
			4	0.1	0.2530	0.2759	0.2654
			4	0.15	**0.3155**	0.2474	**0.3507**
			4	0.2	0.2530	**0.3732**	0.3116
			4	0.25	0.2304	0.2759	0.2984
			4	0.3	0.2398	0.2881	0.2647
			4	0.35	0.2355	0.2689	0.2654
			4	0.4	0.2475	0.2997	0.2449
			4	0.45	0.1304	0.2474	0.1654
			4	0.5	0.1050	0.1504	0.1449

**Table 3:** Statistical validation of the correlation between land-use conversion, climate change and biodiversity change for Landscape based dataset and Number of Tiger reserves based dataset.

Datasets	Nodes	Edges	Correlating criteria	Pearson’s correlation coefficient (r)	
Landscape based distribution	18	70	Temperature anomalies per C_i_	I_2_ I_1_	0.41
				I_3_ I_1_	**0.83**
				I_3_ I_2_	0.82
			Precipitation anomalies per C_i_	I_2_ I_1_	0.41
				I_3_ I_1_	0.58
				I_3_ I_2_	0.47
Number of Tiger Reserves based distribution	18	63	Number of Tiger Reserves per C_i_	I_2_ I_1_	**0.97**
			Avg % increase in core area of Reserve per C_i_	I_2_ I_1_	0.4

As evident from Table 2, modularity based comparative analysis on the two novel datasets proves the superiority of the second dataset based on the number of Tiger reserves; where the second dataset records better performance with maximum modularity values obtained as—binQIEA [0.3155], numQIEA [0.3732] and QDMPSO [0.3507] as compared to binQIEA [0.2068], numQIEA [0.2784] and QDMPSO [0.2787] for Landscape based dataset.

Correspondingly, the modularity distribution for varying values of mixing parameter (µ) for the two novel datasets also validates the above observation; with the second dataset based on the number of Tiger reserves showing better modularity distribution averaged over 40 runs as shown in Fig. 4 a–c.

Thus, the proposed novel implementation of quantum inspired community detection algorithms viz.—binQIEA, numQIEA and QDMPSO and their modularity based comparative analysis with varying values of mixing parameter (µ) on two novel datasets; further cements the superiority of the second dataset based on the number of Tiger reserves by registering better modularity distribution for all the three QIML based CD techniques.

Consequently, the modularity based comparative analysis of QIML v/s std. CD algorithms for the same two novel datasets viz.—landscape based dataset and Number of Tiger reserves based dataset is carried out to validate the improved performance recorded by QIML based CD algorithms for the novel datasets. Out of the six QIML based CD techniques considered for the comparative analysis in^70^, we have taken the most suitable three hybrid algorithms for subsequent modularity based comparison on the two novel datasets; implemented on a classical machine.

**Figure 5:**
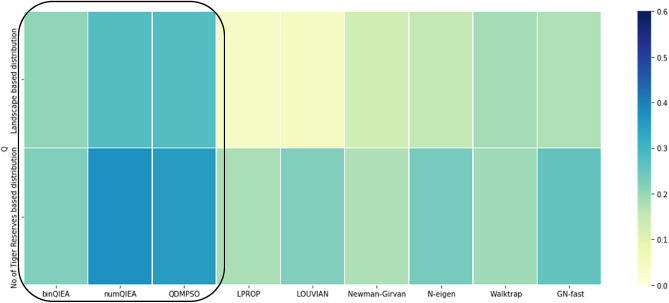
Modularity comparison of QIML versus standard CD techniques for two novel datasets.

As shown in the heatmap given in Fig. 5, QIML based CD community detection algorithms viz.—binQIEA^62,65^, numQIEA^63,65^, QDMPSO^66^ perform better than the standard state of the art algorithms like—LPROP^53^, LOUVIAN^50^, CNM^54^, N-eigen^55^, Walktrap^56^ and GN fast^57^ in terms of the modularity values obtained for the two novel datasets benchmarked for analyzing biodiversity change dynamics. Moreover, out of the two datasets studied, the second dataset viz.—Number of Tiger reserves based dataset is found to perform better than the first dataset by demonstrating a more refined community structure validated by its modularity based comparative analysis using both QIML as well as standard state of the art CD algorithms as shown in Fig. 5.

**Figure 6:**
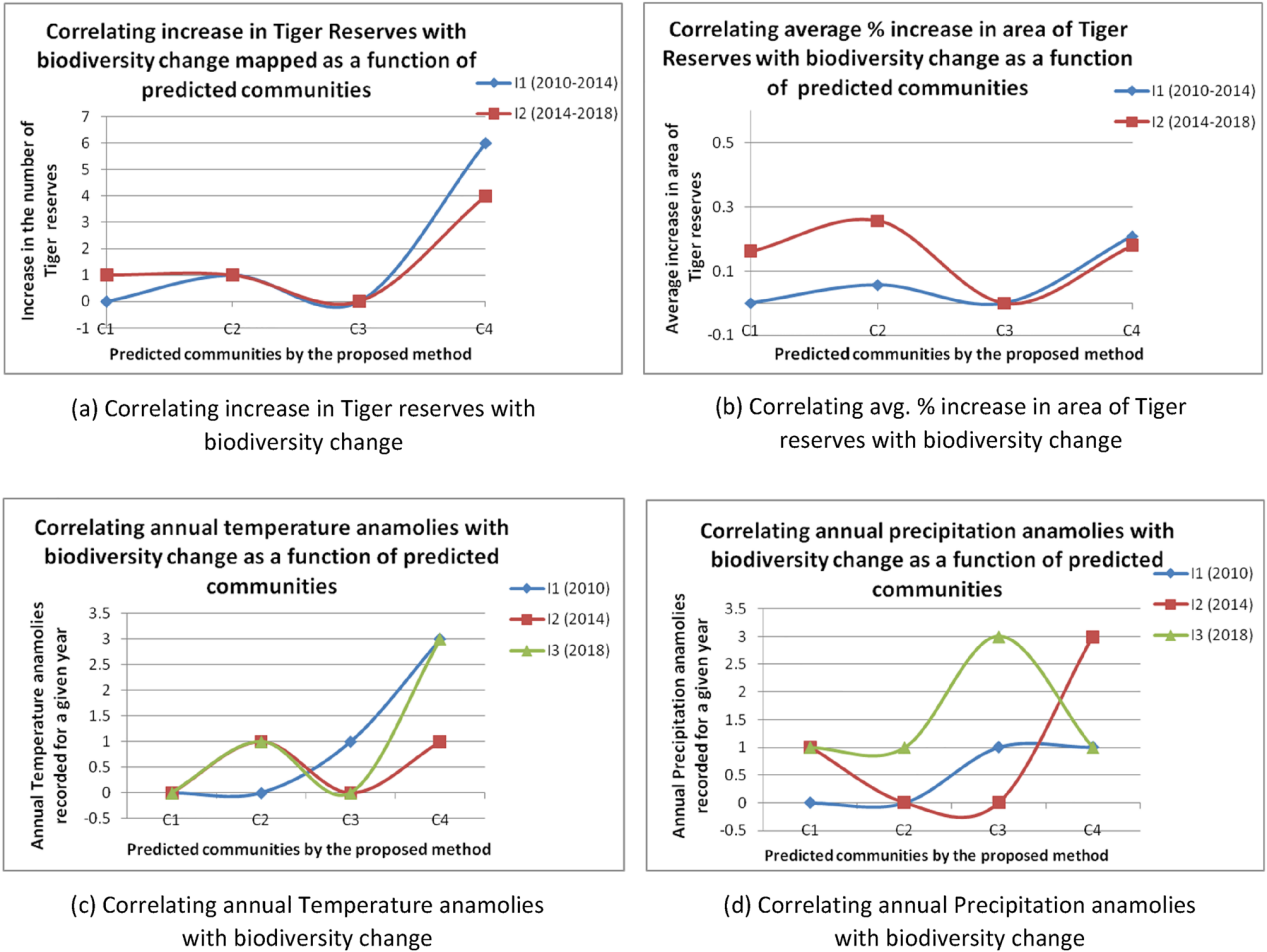
(**a**) and (**b**) Correlating land-use conversion with biodiversity change as a function of four communities predicted by QICD for intervals I_1_ (2010–2014) and I_2_ (2014–2018), (**c**) and (**d**) Correlating climate change with biodiversity change as a function of four communities predicted by QICD for timestamps I_1_ (2010), I_2_ (2014) and I_3_ (2018).

Finally, the statistical validation of the proposed framework designed for analysis of biodiversity change is performed using Pearson’s correlation coefficient and *p* value test. Correlation of land-use conversion with biodiversity change for Number of Tiger Reserves based dataset is plotted as a function of four communities predicted by QICD algorithms (See Fig. 6a,b). Increase in the number of Tiger Reserves and Avg. % increase in core area of Reserves are recorded for intervals I_1_ (2010–2014) and I_2_ (2014–2018) based on data referred from^74^ (Supplementary Table 5).

Similarly, the correlation of climate change with biodiversity change for Landscape based dataset is plotted as a function of four communities predicted by QICD algorithms (See Fig. 6c,d). Temperature anomalies and Precipitation anomalies are recorded for timestamps I_1_ (2010), I_2_ (2014) and I_3_ (2018) based on data referred from^75^ and other reports extracted from https://mausam.imd.gov.in (Supplementary Table 6).

Next, the correlation between I_1_ (2010–2014) and I_2_ (2014–2018) drawn for the Number of Tiger Reserves based dataset as illustrated in Fig. 6a,b, is validated by Pearson’s correlation coefficient as shown in Table 3. Also, the correlation between pairwise sets of I_1_ (2010), I_2_ (2014) and I_3_ (2018) drawn for Landscape based dataset as illustrated in Fig. 6c,d, is validated by Pearson’s correlation coefficient as shown in Table 3.

As evident from Fig. 6, correlation of land-use conversion with biodiversity change for Number of Tiger Reserves based dataset is found to be direct and stronger against the indirect and weaker correlation of climate change with biodiversity change for Landscape based dataset; both plotted as a function of four communities predicted by QICD algorithms. Also, I_1_ (2010) and I_2_ (2014) record best r value (0.83) with *p* value (0.1) in correlating climate change with biodiversity change for Landscape based dataset; while I_1_ (2010–2014) and I_2_ (2014–2018) record the best r-value (0.97) with a *p* value (0.028) in correlating land-use conversion with biodiversity change for Number of Tiger Reserves based dataset as inferred from Table 3, Thus, we infer that not only are biodiversity change, land-use conversion and climate change correlated, but also that the detected correlation is statistically significant.

Thus, Quantum inspired community detection not only provides modularity distribution and comparative modularity analysis with std. CD methods based empirical evaluation for two datasets; but also successfully validates association between biodiversity change, land-use conversion and climate change by Pearson’s correlation coefficient and *p* value test.

Consequently, the species distribution modeling aimed at analysis of different components of biodiversity change could harness the power of QML by either overcoming the computational constraints using quantum devices with classical or quantum algorithms, or by handling uncertainties in associated drivers of biodiversity change like—climate change and land-use conversion and recording improved efficiency by augmenting quantum parallelism with evolutionary algorithms as discussed earlier. Although quantum computers with as many as 2000 qubits have been built, yet its accessibility to most researchers remains an issue. Accordingly, Quantum inspired machine learning (QIML) as a sub-class of QML allows the use of quantum inspired algorithms that could be run on classical computers. Thus, the latter has been found to be used more explicitly as yet in quantum machine learning based algorithms. Properties like—quantum parallelism, absence of the need to prior decide cluster size and architecture, etc. have made QIML based CD algorithms one of the most suitable class of algorithms to be used for the analysis of biodiversity change in the coming future.

## Conclusion and future scope

The proposed novel implementation of quantum inspired machine learning (QIML) based CD algorithms viz.—QIEA bin, QIEA num and QDMPSO to analyze Tiger population in India led to benchmarking of two novel datasets (Landscape based and Number of tiger reserves based datasets). This was followed by their empirical validation based on standard CD metrics like—degree centrality, edge-betweenness centrality and degree distribution. Accordingly, the benchmarked datasets are further evaluated based on the most popularly used performance measures like—modularity, NMI and ARI using Girvan Newman algorithm as a standard CD approach. Lastly, QIML based CD algorithms are implemented—to obtain modularity based distribution to compare the two datasets and for modularity based comparative analysis with Std. CD methods to cement the superiority of the proposed QICD approach for analysis of biodiversity change.

Consequently, both performance measures and centrality measures based comparison has revealed the better of the two datasets; as the number of Tiger reserves based dataset is found to deliver a more defined and centralized community structure based on the given criteria. Modularity based comparative analysis of QIML based CD algorithms with the existing state of the art CD algorithms on the same datasets further validated the supremacy of QIML algorithms over the standard state of the art CD algorithms. Additionally, modularity distribution based on parameter tuning obtained using QIML based CD algorithms also establishes the superiority of the second dataset based on the number of Tiger reserves—in predicting more important nodes and recording higher centralization of nodes.

Lastly, Pearson’s correlation coefficient and *p* value test are introduced for statistical validation of correlations established between biodiversity change and land-use conversion or climate change. It clearly establishes how land-use conversion and climate change are two of the many drivers of biodiversity change; with land-use conversion based habitat change being a direct driver and anomalies in climate (Temp., Prec., etc.) being an indirect driver of biodiversity change.

As a future initiative, other centrality measures could also be used like—closeness centrality and eigenvector centrality^35^. Performance evaluation using measures like—purity^42^, fuzzy rand index^43^, etc. could also be used for overlapping as well as disjoint communities. Moreover, community detection approaches could be extended for analysis with other drivers and their interactions; ameliorating or exacerbating biodiversity change. Metrics like Community Temperature Index^44^ and Living Planet Index^45^ could also be explored to study species abundance change in the future. Box-plot based comparative analysis could also be done to assess computational time for proposed implementations of CD algorithms.

Thus, the formulation of strategies and reforms to contain biodiversity change needs special focus to realize the goals of sustainable development. Hybridization of machine learning techniques with quantum algorithms could completely revolutionalize the analysis of biodiversity change in the coming time.

## Supplementary Information

Supplementary Information.

## Data Availability

All data generated or analysed during this study are included in this published article (and its Supplementary Information files).
