# Supplementary material for: Quantum inspired community detection for analysis of biodiversity change driven by land-use conversion and climate change
Source: Sci Rep. 2021 Jul 12;11:14332. doi: 10.1038/s41598-021-93122-x (PMC8275618; doi:10.1038/s41598-021-93122-x)
Supplement: Supplementary file 1 — Supplementary Information. [file 41598_2021_93122_MOESM1_ESM.pdf]

**Supplemental Material**

Supplement to: Sana Akbar, Sri Khetwat Saritha. Quantum inspired community detection for analysis of biodiversity change driven by land-use conversion and climate change

## Table of Contents

Supplementary Figure 1. Components of biodiversity change....................................................................3

Supplementary Figure 2. Classification of climate change factors ………................................................4

Supplementary Table 1. State-wise Tiger population in India for the years 2010, 2014 and 2018………...5

Supplementary Table 2. Division of 18 states into 4 subgroups based on percentage increase in Tiger population in India [2010 to 2014] ………………………………………………………………….... 6

Supplementary Table 3. State-wise list of Tiger reserves in India………………………………………… 7

Supplementary Table 4. Division of 18 states into 2 subgroups based on the number of Tiger Reserves in each state……………………………………………………………………………………….…...8

Supplementary Figure 3. Modularity based comparative analysis of the proposed novel implementation of quantum inspired community detection algorithms against the std. CD approaches.................................. 9

Supplementary Figure 4. Evolution of Quantum inspired machine learning from data science................ 10

Supplementary Figure 5. Hierarchial bi-partitioning using QICD algorithm ……………………………11

Supplementary Figure 6. Proposed framework for analysis of biodiversity change……………………... 12

Supplementary Figure 7(a) and Fig.7 (b). Network diagrams for Landscape based dataset and Number of Tiger reserves based dataset (drawn using igraph package in R studio)……………………………….... 13

(Supplementary Fig. 8(a) and Fig.8 (b)). Communities detected for Landscape based dataset and Number of Tiger reserves based dataset respectively (drawn using Netdraw package)…………….…………….…...14

Supplementary Table 5. Correlating land-use conversion with biodiversity change as a function of four communities predicted by QICD………………………………………………………......................... 15

Supplementary Table 6. . Correlating climate change with biodiversity change as a function of four communities predicted by QICD………………………………………………………………………. 16

**Supplementary Figure 1. Components of biodiversity change.** The two components of biodiversity change are illustrated with their four dimensions including - species extinctions, species abundances, species distributions, and genetic diversity.

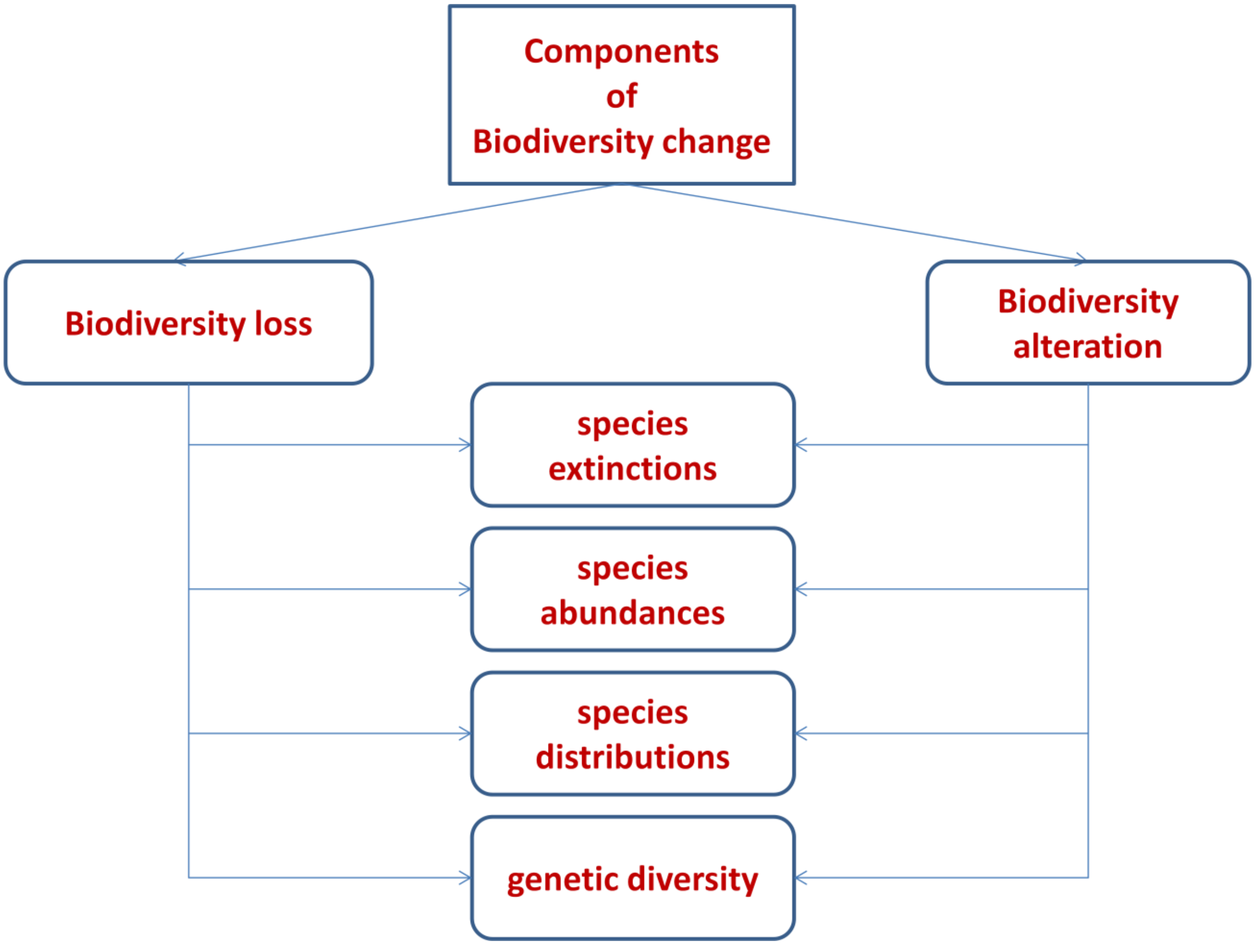

**Supplementary Figure 2. Classification of climate change factors.** Climate change is categorized as natural or anthropogenic. Natural climate change is further characterized as a response to - a number of *internal or external factors* and *internal climate change variability* as shown in Fig. 1.3. Factors like- change in earth's orbital parameters or solar luminosity, volcanic eruptions, change in earth's geographical state or atmospheric composition together constitute the *internal and external factors*. On the contrary, natural fluctuations demonstrating strongly nonlinear characteristics that occur despite constant external conditions like - periodic self-sustained climatic oscillations or large scale atmospheric circulation leading to cyclones and anti-cyclones; contribute to *internal climate change variability. Anthropogenic climate change* on the other hand is an outcome of human activities like – emission of greenhouse gases, aerosols, etc. and land cover changes.

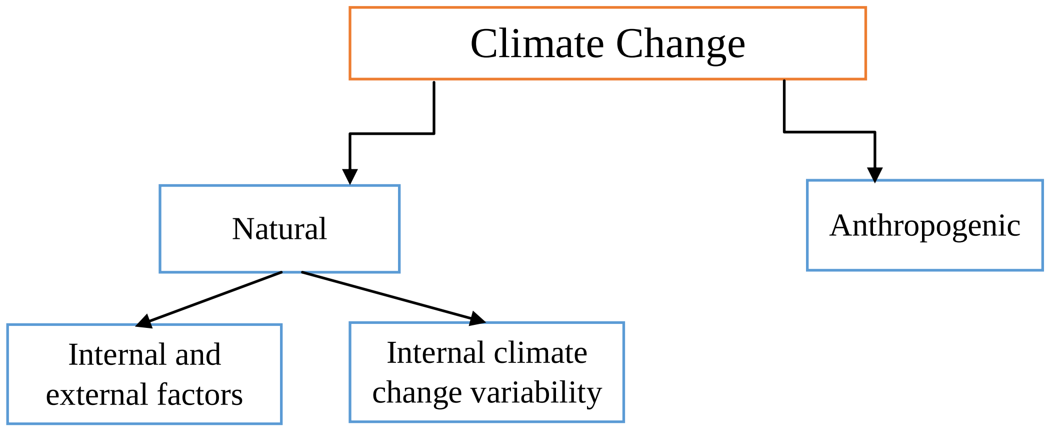

**Supplementary Table 1. State-wise Tiger population in India for the years 2010, 2014 and 2018.**
[*"India's tiger population sees 33% increase"*, *BBC. 29 July 2019.* https://www.bbc.com/news/world-asia-india-49148174 and [https://bigcatsindia.com/tiger-census-2018/.]

| Tiger population growth in India | | | |
|---|---|---|---|
| **State** | **2010** | **2014** | **2018** |
| **Shivalik Gangetic Plain** | | | |
| Uttarakhand | 227 | 340 | 442 |
| Uttar Pradesh | 118 | 117 | 173 |
| Bihar | 8 | 28 | 31 |
| **Central Indian Landscape and Eastern Ghats** | | | |
| Andhra Pradesh & Telangana | 72 | 68 | 48 & 26 |
| Chhattisgarh | 26 | 46 | 19 |
| Madhya Pradesh | 257 | 308 | 526 |
| Maharashtra | 169 | 190 | 312 |
| Odisha | 32 | 28 | 28 |
| Rajasthan | 36 | 45 | 69 |
| Jharkhand | 10 | 3 | 5 |
| **Western Ghats** | | | |
| Karnataka | 300 | 406 | 524 |
| Kerala | 71 | 136 | 190 |
| Tamil Nadu | 163 | 229 | 264 |
| Goa | – | 5 | 3 |
| **The Northeast** | | | |
| Assam | 143 | 167 | 190 |
| Arunachal Pradesh | – | 28 | 29 |
| Mizoram | 5 | 3 | 0 |
| Northern West Bengal | – | 3 | 0 |
| Sundarbans | 70 | 76 | 88 |
| **Total** | **1,706** | **2,226** | **2967** |

**Supplementary Table 2. Division of 18 states into 4 subgroups based on percentage increase in Tiger population in India [2010 to 2014].** Amongst the eighteen states surveyed in 2014, the most favorable region comprising of three states recorded the highest increase (above 40%), while the new additions constituted by two states; represented areas that bred tigers for the first time. The favorable region formed by eight states registered an average performance (10-40%) while the least favorable region consisting of five states reported a decline in tiger population.

| ***Most favorable states*** (% increase in Tiger population: above 40%) | ***Newly added states*** (% increase in Tiger population: above 100%) | **Favorable states** (% increase in Tiger population: (10-40%)) | **Least favorable states** (% increase in Tiger population: (less than 0)) |
|---|---|---|---|
| Bihar | Arunachal Pradesh | Uttarakhand | Uttar Pradesh |
| Chhattisgarh | West Bengal | Maharashtra | Andhra Pradesh |
| Kerala | | Madhya Pradesh | Odisha |
| | | Rajasthan | Jharkand |
| | | Karnataka | Mizoram |
| | | Tamil Nadu | |
| | | Assam | |
| | | Sunderbans | |

**Supplementary Table 3. State-wise list of Tiger reserves in India [**https://bigcatsindia.com/tiger-census-2018/.]

| S.N. | Name of Tiger Reserve | State | S.N. | Name of Tiger Reserve | State |
|---|---|---|---|---|---|
| 1 | Nagarjunsagar Srisailam | Andhra Pradesh | 27 | Melghat | Maharashtra |
| 2 | Namdapha | Arunachal Pradesh | 28 | Tadoba-Andhari | Maharashtra |
| 3 | Kamlang Tiger Reserve | Arunachal Pradesh | 29 | Pench | Maharashtra |
| 4 | Pakke | Arunachal Pradesh | 30 | Sahyadri | Maharashtra |
| 5 | Manas | Assam | 31 | Nawegaon-Nagzira | Maharashtra |
| 6 | Nameri | Assam | 32 | Bor | Maharashtra |
| 7 | Orang Tiger Reserve | Assam | 33 | Dampa | Mizoram |
| 8 | Kaziranga | Assam | 34 | Similipal | Odisha |
| 9 | Valmiki | Bihar | 35 | Satkosia | Odisha |
| 10 | Udanti-Sitanadi | Chattisgarh | 36 | Ranthambore | Rajasthan |
| 11 | Achanakmar | Chattisgarh | 37 | Sariska | Rajasthan |
| 12 | Indravati | Chhattisgarh | 38 | Mukandra Hills | Rajasthan |
| 13 | Palamau | Jharkhand | 39 | Kalakad-Mundanthurai | Tamil Nadu |
| 14 | Bandipur | Karnataka | 40 | Anamalai | Tamil Nadu |
| 15 | Bhadra | Karnataka | 41 | Mudumalai | Tamil Nadu |
| 16 | Dandeli-Anshi | Karnataka | 42 | Sathyamangalam | Tamil Nadu |
| 17 | Nagarahole | Karnataka | 43 | Srivilliputhur Meghamalai | Tamil Nadu |
| 18 | Biligiri Ranganatha Temple | Karnataka | 44 | Kawal | Telangana |
| 19 | Periyar | Kerala | 45 | Amrabad | Telangana |
| 20 | Parambikulam | Kerala | 46 | Dudhwa | Uttar Pradesh |
| 21 | Kanha | Madhya Pradesh | 47 | Pilibhit | Uttar Pradesh |
| 22 | Pench | Madhya Pradesh | 48 | Amangarh (buffer of Corbett TR) | Uttar Pradesh |
| 23 | Bandhavgarh | Madhya Pradesh | | Corbett | Uttarakhand |
| 24 | Panna | Madhya Pradesh | 49 | Rajaji TR | Uttarakhand |
| 25 | Satpura | Madhya Pradesh | 50 | Sunderbans | West Bengal |
| 26 | Sanjay-Dubri | Madhya Pradesh | 51 | Buxa | West Bengal |

**Supplementary Table 4. Division of 18 states into 2 subgroups based on the number of Tiger Reserves in each state.**

| S.No. | State | Number of Tiger Reserves |
|---|---|---|
| 1 | Andhra Pradesh | 1 |
| 2 | Arunachal Pradesh | 2 |
| 3 | **Assam** | **3** |
| 4 | Bihar | 1 |
| 5 | **Chattisgarh** | **3** |
| 6 | Jharkhand | 1 |
| 7 | **Karnataka** | **4** |
| 8 | Kerala | 2 |
| 9 | **Madhya Pradesh** | **6** |
| 10 | **Maharashtra** | **5** |
| 11 | Mizoram | 1 |
| 12 | Odisha | 2 |
| 13 | **Rajasthan** | **3** |
| 14 | **Tamil Nadu** | **4** |
| 15 | Uttar Pradesh | 2 |
| 16 | Uttarakhand | 2 |
| 17 | Sunderbans | 1 |
| 18 | West Bengal | 1 |

**Supplementary Figure 3. Modularity based comparative analysis of the proposed novel implementation of quantum inspired community detection algorithms against the std. CD approaches [50, 53-57].** It is carried out for *the same two novel datasets* viz. - landscape based and Number of Tiger reserves based dataset to validate the improved performance recorded by QIML based CD algorithms for the novel datasets. Out of the six QIML based CD techniques considered for the comparative analysis in [70]; we have taken the best three for subsequent modularity based comparison on the two novel datasets.

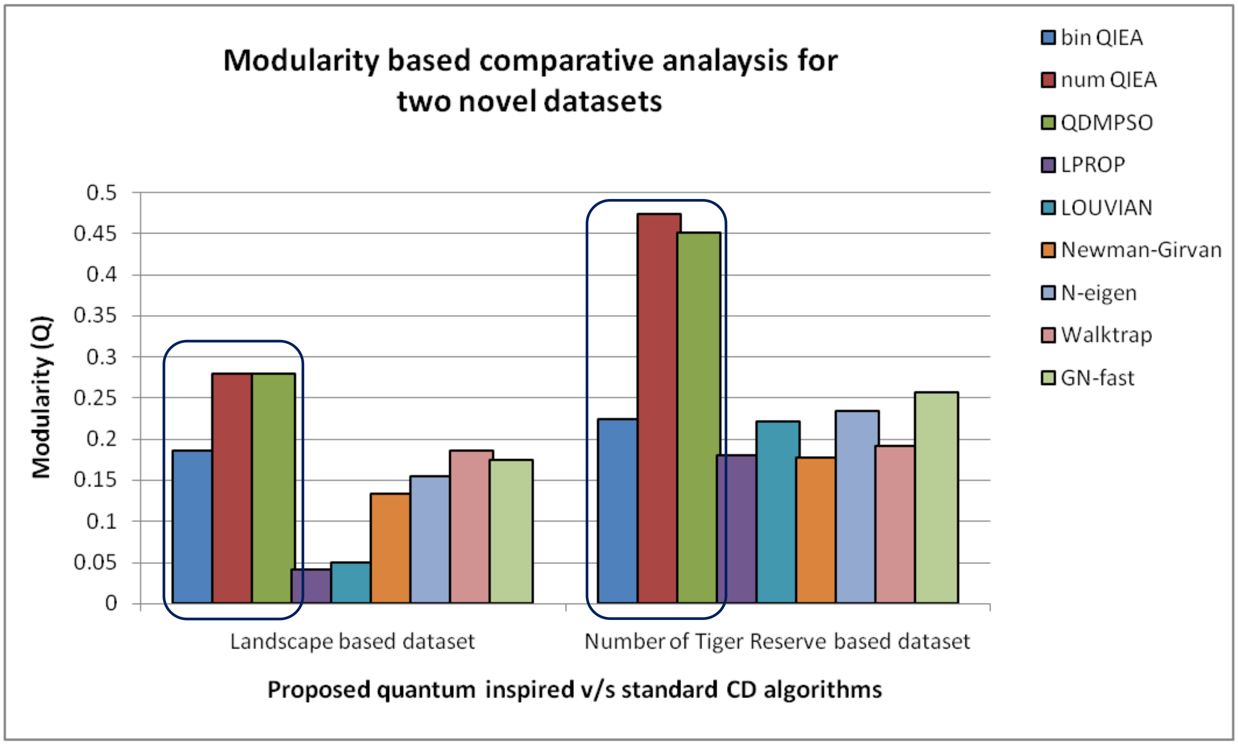

**Supplementary Figure 4. Evolution of Quantum inspired machine learning from data science.** QIML could thus be inferred to have evolved from QML; branching out of QIPR (Quantum Information Processing Research), which could be viewed as a novel prospect of IR (Information Retrieval); being an indispensable part of data science.

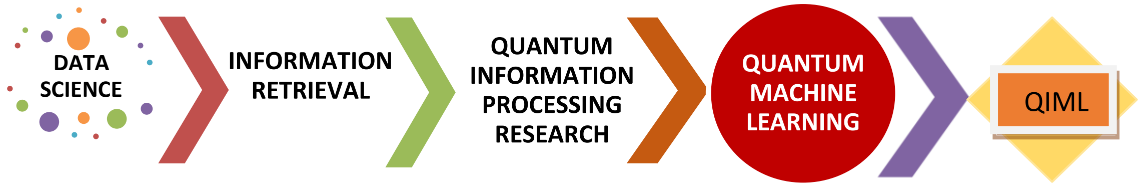

**Supplementary Figure 5. Hierarchial bi-partitioning using QICD algorithm.** A typical QIEA starts with the initialization of a *quantum population* consisting of n qubits forming a *quantum chromosome*; with n denoting the no. of nodes in the network. Accordingly, both Landscape and number of Tiger reserves based datasets are represented by a chromosome of length n=18 as it consists of 18 nodes; constituting 70 and 63 edges respectively. The *measure operation;* when applied to the quantum chromosome, renders it into a string of classical binary bits forming P(t). The first level depicts division into two communities represented by 0 and 1, followed by division of 0 into (0 and 2) and 1 into (1 and 3) leading to the formation of four communities at the second level. This goes on until no new division is possible.

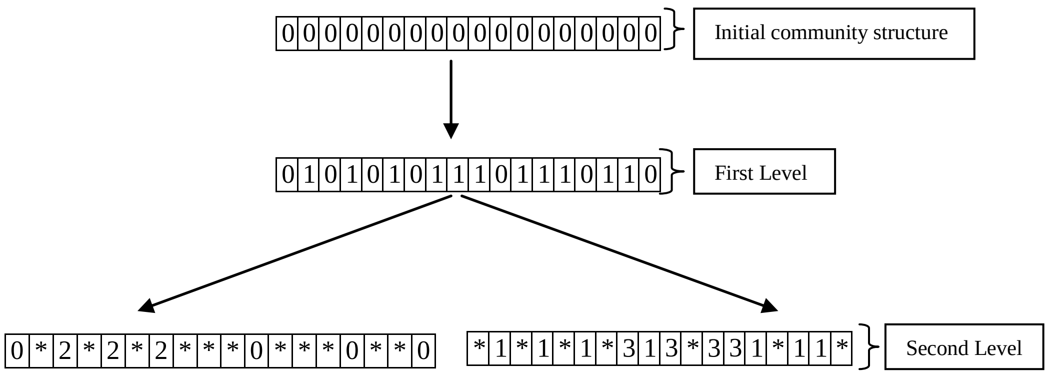

**Supplementary Figure 6. Proposed framework for analysis of biodiversity change**

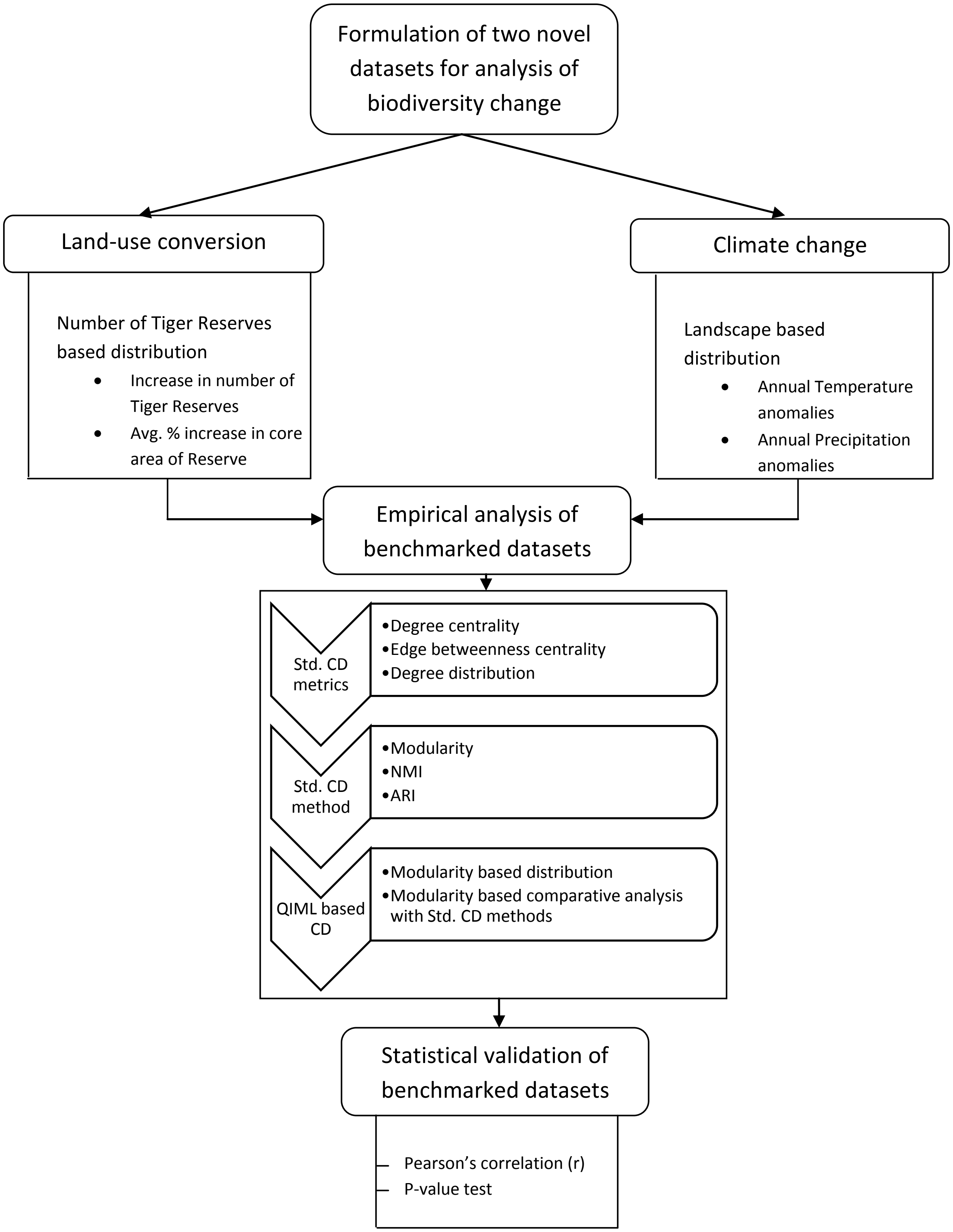

**Supplementary Figure 7(a) and Fig.7 (b). Network diagrams for Landscape based dataset and Number of Tiger reserves based dataset (drawn using igraph package in R studio).**

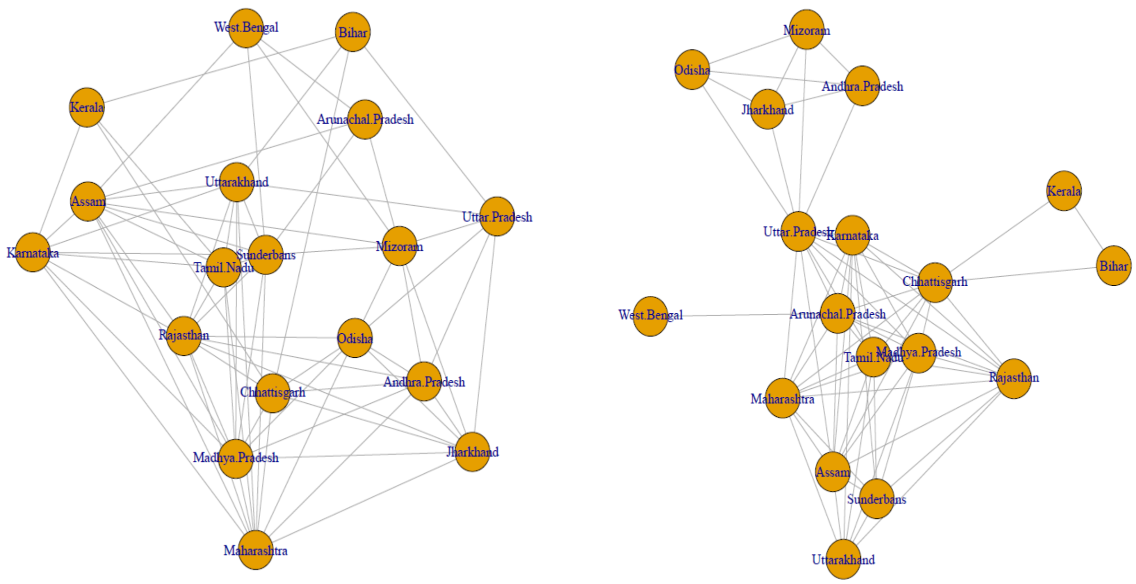


(a) Landscape based dataset

(b) Number of Tiger reserves based dataset

**(Supplementary Fig. 8(a) and Fig.8 (b)). Communities detected for Landscape based dataset and Number of Tiger reserves based dataset respectively (drawn using Netdraw package).** We have used Girvan Newman as the most standardized community detection algorithm to validate the two novel datasets by comparative analysis of different performance measures like- modularity (Q), NMI and ARI used for both datasets. Graph partitioning for division into communities using this method is done by calculating the edge-betweenness of each node, followed by removal of the edge with the highest betweenness. If this leads to separate sub-graphs, the graph is partitioned into two communities. This could go on until no improvement in modularity is possible [35].

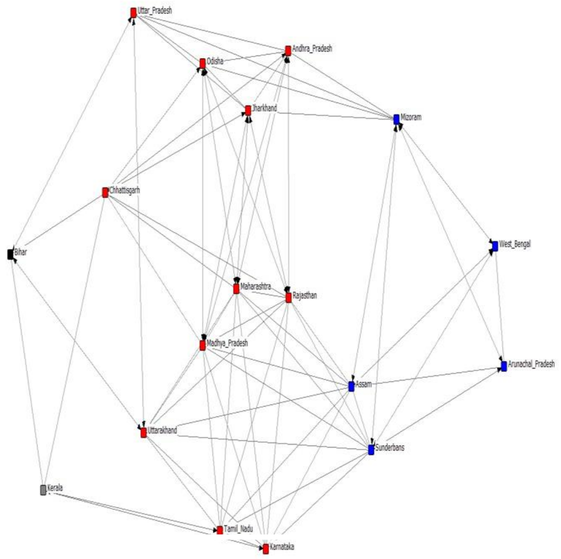


(a) Landscape based dataset

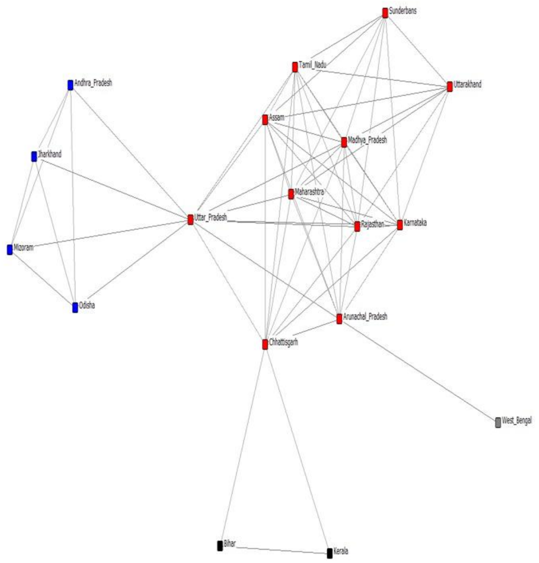


(b) Number of Tiger reserves based dataset

**Supplementary Table 5. Correlating land-use conversion with biodiversity change as a function of four communities predicted by QICD**

| DATASET | $I_1$ (2010-2014) | | | $I_2$ (2014-2018) | | |
|---|---|---|---|---|---|---|
| | States with newly added Tiger Reserves | Belonging to Community $C_i$ | Number of Tiger Reserves per $C_i$ | States with newly added Tiger Reserves | Belonging to community $C_i$ | Number of Tiger Reserves per $C_i$ |
| Number of Tiger Reserves based distribution | Karnataka | $C_4$ | $C_1 \rightarrow 0$ | Uttarakhand | $C_4$ | $C_1 \rightarrow 1$ |
| | Tamil Nadu (2) | | | Tamil Nadu | | |
| | Rajasthan | | $C_2 \rightarrow 1$ | Assam | | $C_2 \rightarrow 1$ |
| | Maharashtra | | $C_3 \rightarrow 0$ | Maharashtra | | $C_3 \rightarrow 0$ |
| | Kerala | | $C_4 \rightarrow 6$ | Arunachal Pradesh | $C_1$ | $C_4 \rightarrow 4$ |
| | Uttar Pradesh | $C_2$ | | Uttar Pradesh | $C_2$ | |
| | States with newly added Tiger Reserves | Belonging to Community $C_i$ | Avg. % increase in core area of Reserve per $C_i$ | States with newly added Tiger Reserves | Belonging to community $C_i$ | Avg. % increase in core area of Reserve per $C_i$ |
| | Karnataka | $C_4$ | $C_1 \rightarrow 0$ | Uttarakhand | $C_4$ | $C_1 \rightarrow 21.2$ |
| | Tamil Nadu (2) | | | Tamil Nadu | | |
| | Rajasthan | | $C_2 \rightarrow 6.9$ | Assam | | $C_2 \rightarrow 33.9$ |
| | Maharashtra | | $C_3 \rightarrow 0$ | Maharashtra | | $C_3 \rightarrow 0$ |
| | Kerala | | $C_4 \rightarrow 25.4$ | Arunachal Pradesh | $C_1$ | $C_4 \rightarrow 23.5$ |
| | Uttar Pradesh | $C_2$ | | Uttar Pradesh | $C_2$ | |

**Supplementary Table 6. Correlating climate change with biodiversity change as a function of four communities predicted by QICD**

| DATASET | $I_1$ (2010) | | | $I_2$ (2014) | | | $I_3$ (2018) | | |
|---|---|---|---|---|---|---|---|---|---|
| Landscape based distribution | States showing Temperature anomaly | Belonging To Community $C_i$ | Temperature anomalies per $C_i$ | States showing Temperature anomaly | Belonging to community $C_i$ | Temperature anomalies per $C_i$ | States showing Temperature anomaly | Belonging to community $C_i$ | Temperature anomalies per $C_i$ |
| | West Bengal | $C_3$ | $C_1 \rightarrow 0$ | Rajasthan | $C_4$ | $C_1 \rightarrow 0$ | Bihar | $C_2$ | $C_1 \rightarrow 0$ |
| | Chhattisgarh | $C_4$ | $C_2 \rightarrow 0$ | Bihar | $C_2$ | $C_2 \rightarrow 1$ | Rajasthan | $C_4$ | $C_2 \rightarrow 1$ |
| | Uttarakhand | | $C_3 \rightarrow 1$ | | | $C_3 \rightarrow 0$ | Uttar Pradesh | | $C_3 \rightarrow 0$ |
| | Uttar Pradesh | | $C_4 \rightarrow 3$ | | | $C_4 \rightarrow 1$ | Madhya Pradesh | | $C_4 \rightarrow 3$ |
| | States showing Precipitation anomaly | Belonging To Community $C_i$ | Precipitation anomalies per $C_i$ | States showing Precipitation anomaly | Belonging to community $C_i$ | Precipitation anomalies per $C_i$ | States showing Precipitation anomaly | Belonging to community $C_i$ | Precipitation anomalies per $C_i$ |
| | West Bengal | $C_3$ | $C_1 \rightarrow 0$ | Andhra Pradesh | $C_1$ | $C_1 \rightarrow 1$ | Kerala | $C_1$ | $C_1 \rightarrow 1$ |
| | Uttar Pradesh | $C_4$ | $C_2 \rightarrow 0$ | Madhya Pradesh | $C_4$ | $C_2 \rightarrow 0$ | Bihar | $C_2$ | $C_2 \rightarrow 1$ |
| | | | $C_3 \rightarrow 1$ | Uttar Pradesh | | $C_3 \rightarrow 0$ | Assam | $C_3$ | $C_3 \rightarrow 3$ |
| | | | $C_4 \rightarrow 1$ | Maharashtra | | $C_4 \rightarrow 3$ | West Bengal | | $C_4 \rightarrow 1$ |
| | | | | | | | Arunachal Pradesh | | |
| | | | | | | | Maharashtra | $C_4$ | |
